# Morphology of Anterolateral Ligament of the Knee: A Cadaveric Observation with Clinical Insight

**DOI:** 10.1155/2016/9182863

**Published:** 2016-10-25

**Authors:** Bhagath Kumar Potu, Abdel Halim Salem, Marwan F. Abu-Hijleh

**Affiliations:** ^1^Department of Anatomy, College of Medicine & Medical Sciences, Arabian Gulf University, P.O. Box 22979, Manama, Bahrain; ^2^Department of Basic Medical Sciences, College of Medicine, Qatar University, P.O. Box 2713, Doha, Qatar

## Abstract

*Background*. The morphology and function of anterolateral ligament (ALL) of the knee are not clearly understood even today with all the sophisticated techniques available. There have been differing descriptions of the ALL of the knee in literature, and not all of them have been named or described clearly.* Aim*. The present study was undertaken to provide a clear structure/relationship description on ALL.* Materials and Methods*. We used 24 formalin-fixed cadaveric limbs. Knee regions of the all the limbs were neatly dissected and the ALL was exposed. Its proximal and distal attachments were traced carefully. Middle portion of ALL was removed and processed for histological analysis.* Results*. ALL was found in one right knee (4.16%). It extended distally from the lateral femoral condyle to the lateral tibial plateau margin. Its attachment on the tibial plateau was located between head of the fibula and Gerdy's tubercle. A strong connection was identified between the ALL and the periphery of the middle third of the lateral meniscus. Histological analysis of ALL confirmed the presence of true ligamentous structure in it with dense connective tissue and plenty of fibroblasts.* Conclusion*. The prevalence of ALL in different populations along with its clinical significance has been discussed in detail in this paper.

## 1. Introduction

The tibiofemoral articulation of the human knee joint is stabilized from either side by medial and lateral collateral ligaments. While this traditional description goes on in anatomical textbooks, a significant debate sprouted on accessory structures of the lateral knee to understand their functions [1]. One such accessory ligament that was described in recent orthopedic research is the anterolateral ligament (ALL) [2]. From cadaveric dissections, it is reported that the ALL takes its origin from the lateral femoral condyle with the lateral collateral ligament (LCL); it then runs obliquely deep to the iliotibial tract (ITT) to be inserted on midportion of proximal tibia between Gerdy's tubercle and fibular head [2]. It was Segond, a French surgeon, who first described this structure as a fibrous band [3]. After a historical long gap in the literature, it was Hughston et al. [4] who described this structure as the lateral capsular ligament. This finding was subsequently supported by many studies and derived the same name [5–8]. Campos et al. [9] described this structure as an anterior oblique band. The various terms depicting the ALL have led to much confusion about the precise anatomy and function of this structure. This might explain why classical anatomical textbooks preferred not to mention this unique structure. The name “ALL” has appeared in literature very recently by Vieira et al. [10] and Vincent et al. [11]. All the above-mentioned terms are now been standardized as ALL by Claes et al. [2]. The purpose of this study was to have a detailed anatomical characterization of the ALL from an available small cadaveric sample. The morphology and clinical background of ALL are very important for the anatomists and orthopedic surgeons to keep in mind during their respective practices. We have discussed detailed morphology and clinical significance of ALL in this paper.

## 2. Material and Methods

In the process of understanding the anatomy of the knee through dissections, we have conducted a cadaveric study (cadavers were imported from Europe of Caucasian origin) on 24 formalin-fixed knees (11 right and 13 left) at Anatomy and Pathology Learning Resource Centre (APLRC) of the Arabian Gulf University, College of Medicine. Study was conducted from August 2014 to June 2015 and it was approved by the Research and Ethics Committee. The gender and age of the dissected knees are not known.

### 2.1. Dissection Protocol

Dissection was started on the lateral aspect of the flexed knee. The iliotibial tract (ITT) was identified and was subsequently cut to visualize the fibrous capsule of knee joint. The lateral collateral ligament (LCL) was then palpated with the knee in slight varus. The fibers of the lateral ligament were carefully traced to identify ALL if present. Where ALL was present, it was traced to determine its origin, insertion, and interconnecting fibers with the lateral meniscus.

A digital caliper was used to measure the length, width of the ALL at femoral origin and tibial insertion, and width-thickness of the ALL at the level of the knee joint line.

After measurements, specimens were obtained for evaluating the ligament's microscopic structure. The ALL was sectioned by standard protocol as described below.

### 2.2. Histological Analysis

The entire harvested specimen was fixed in 10% formaldehyde. The specimens were embedded in paraffin, and 4 *μ* sections were processed and stained with Masson's trichrome staining as per standard protocol [18]. Longitudinal sections of the ligament were evaluated to study the organization of collagen fibers in the ligament.

## 3. Results

**Figure 1:**
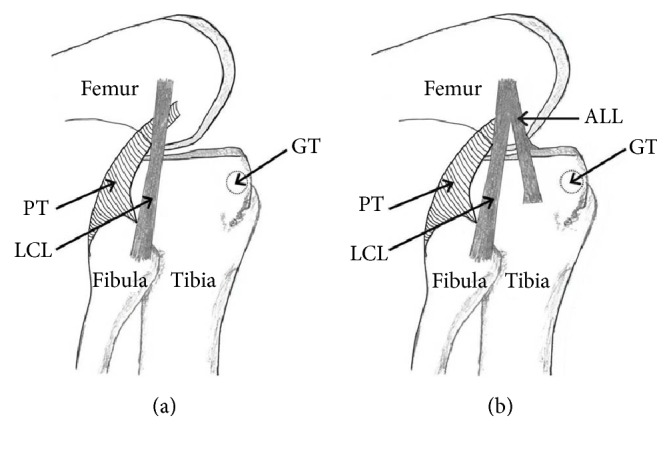
(a) Schematic drawing showing the LCL with no ALL present on the lateral knee. ALL: anterolateral ligament; LCL: lateral collateral ligament; PT: popliteus tendon; GT: Gerdy's tubercle. (b) Schematic drawing showing the ALL (its proximal bifurcation from LCL) and its important relations on the lateral knee. ALL: anterolateral ligament; LCL: lateral collateral ligament; PT: popliteus tendon; GT: Gerdy's tubercle.

**Figure 2:**
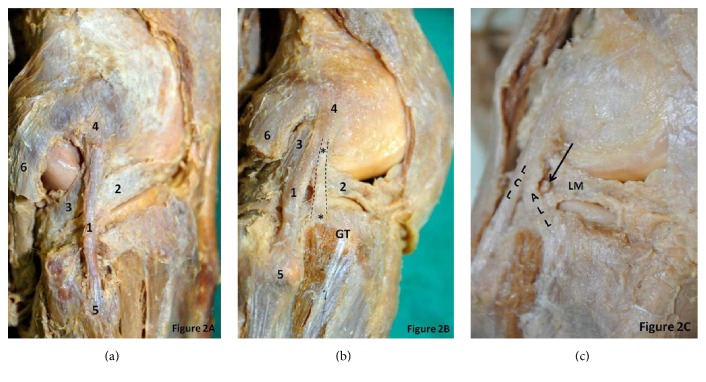
(a) Dissection of the right knee showing LCL with no ALL and its important relations on the lateral knee. 1: lateral collateral ligament; 2: lateral meniscus; 3: popliteus tendon; 4: lateral condyle of femur; 5: head of fibula; 6: lateral head of gastrocnemius; GT: Gerdy's tubercle. (b) Dissection of the right knee showing ALL (taking its proximal bifurcation from LCL) and its important relations on the lateral knee. *∗*: anterolateral ligament; 1: lateral collateral ligament; 2: lateral meniscus; 3: popliteus tendon; 4: lateral condyle of femur; 5: head of fibula; 6: lateral head of gastrocnemius; GT: Gerdy's tubercle. Dotted lines outline the extent of ALL. (c) Superficial dissection of right knee showing lateral geniculate vessels situated between ALL and LM. ALL: anterolateral ligament; LCL: lateral collateral ligament; LM: lateral meniscus. *∗* showing the lateral geniculate vessels.

ALL was found to be present only in 1 right knee (4.16%) out of 24 specimens that were dissected (Figure 2(b)). When ITT was removed, the soft tissues overlying the lateral femoral condyle were seen clearly. ALL was observed outside the capsule of the joint, extending from the lateral femoral condyle to the tibial plateau and lateral meniscus. Its attachment to tibial plateau was located between head of the fibula and Gerdy's tubercle (Figure 1(b)). In view of its location, appearance, and orientation, the observed structure was confirmed as ALL. The ALL measurements obtained are presented in Table 1.

**Figure 3:**
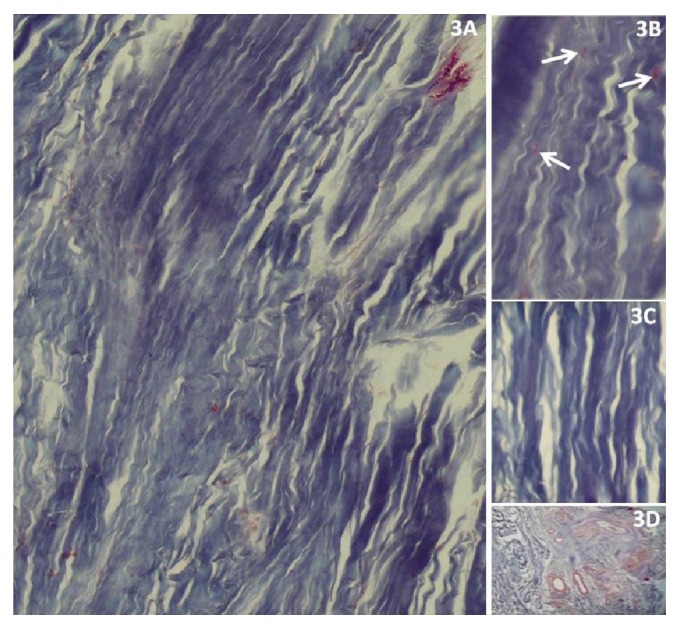
Histological sections of the ALL demonstrate dense collagen fibers (3A: Masson's trichrome staining at magnification of 20x; 3C: Masson's trichrome staining at magnification of 40x); fibroblasts (arrows in 3B: Masson's trichrome staining at magnification of 40x); vascular tissue (3D: Masson's trichrome staining at magnification of 10x).

Histological analysis using Masson's trichrome stained sections revealed that the ligament has dense connective tissue in the form of collagen bundles with fibroblasts and vascular tissue in it (Figures 3A–3D).

## 4. Discussion

The key finding in present study is the presence of ALL at anterolateral aspect of the human knee and this is found to be made up of dense collagen fibers (Figure 1(b)). The morphometry of ALL in our study seems to have same morphometry as reported in recent literature [2] (Table 1). Most of the recent studies found the existence of ALL presence in all cases. Cadaveric, surgical, and imaging studies from the literature report the prevalence of ALL ranging within 20–100% in various populations (Table 2). Such reported prevalence was found to be agreeable with our findings. This could be due to genetic factors and ethnic diversity and also due to the fact is that our cadaveric sample was of small size. From our cadaveric dissection, it is apparent that ALL takes its origin from the lateral femoral condyle with the LCL (Figure 2(b)). It runs obliquely parallel but deep to the iliotibial tract (ITT). It is inserted on the midportion of the proximal tibia halfway between Gerdy's tubercle and the fibular head.

We have observed in our study that the lateral inferior geniculate artery and vein are situated between the lateral meniscal rim and the ALL (Figure 2(c)) at the level of the joint line as reported earlier [2, 10, 11]. A study by Claes et al., and an MRI study by Helito et al., discussed that the relationship of the lateral inferior geniculate vessels with ALL is an important anatomical landmark useful in identifying ALL. The histological analysis of ALL revealed dense connective tissue and fibroblasts suggestive of a true ligament and this confirms that it is not just a capsular thickening (Figures 3A–3D). It has been reported that when ALL is present, it is attached to the peripheral portions of lateral meniscus. We also have observed the same findings in our case and our observation agrees with the study performed by Helito et al. where ALL was inserted to the outer diameter of the menisci. Thus, it is possible to assume that the ALL may result in the lateral meniscus tears, in particular peripheral rim detachment tears [2]. In an MRI study by Van Dyck et al. [19], 41 out of 90 knees demonstrated ALL abnormalities, and, with the remaining 49 knees with intact ALL, 15 had a torn lateral meniscus as compared to 25 lateral menisci of 41 knees with abnormal ALL. Thus, this study supports the hypothesis that the ALL injuries are relatively common and also associated with lateral menisci tears. While Van Dyck et al. [19] findings are as mentioned above, Helito et al. reported an MRI study on abnormalities of ALL and found that there is no relationship between ALL and lateral menisci injuries. The ALL may play a role in the prevention of anterior tibial translation because of its close attachment to Gerdy's tubercle (GT). Thus, its preservation may be important to avoid excessive anterior tibial translation following total knee arthroplasty. One of the limitations of this study is that the sample size is small and we do not know the gender and age of the dissected knees. Nevertheless, this study is significant since ALL is present in only one of the cases we dissected.

## 5. Conclusion

This study provides a morphological description of the ALL in small cadaveric sample and discusses its prevalence in different populations. Study of ALL opens up a new line of research for orthopedic surgeons and radiologists and the awareness on ALL might help sports physicians, physiotherapists, and orthopedic surgeons in identifying and treating injuries of lateral side of the knee.

## Figures and Tables

**Table 1:** Summarizing the length, width, thickness, and distance of the anterolateral ligament (ALL) from Gerdy's tubercle (GT).

ALL length (mm)		ALL width (mm)			ALL thickness (mm)	Distance between ALL and GT (mm)
Extension	Flexion (90)	Femoral origin	Joint line	Tibial insertion	Joint line	15.06
34.23	30.41	4.83	4.04	6.06	1.78	

**Table 2:** Showing cadaveric studies and their ALL prevalence reported in literature.

Studies	Population	Number of knees	Name	Prevalence
Campos et al., [9]	American	6	Anterior oblique band	6 (100%)
Claes et al., [2]	Belgian	41	ALL	40 (98%)
Diamantopoulos et al., [12]	Greek	10	Anatomic variation of LCL	2 (20%)
Dodds et al., [13]	British	40	ALL	33 (82.5%)
El-Gharbawy [14]	Egyptian	10	Tibial band of LCL	2 (20%)
Helito et al., [15]	Brazilian	6	ALL	6 (100%)
Helito et al., [16]	Brazilian	20	ALL	20 (100%)
Irvine et al., [6]	British	7	Anterior oblique band	7 (100%)
Johnson [5]	American	6	Lateral capsular ligament	6 (100%)
Monaco et al., [17]	Italian	6	Anterolateral femorotibial ligament	6 (100%)
Vincent et al., [11]	French	10	ALL	10 (100%)
Our study	Caucasian	24	ALL	1 (4.16%)

## References

[1] Sanchez A. R., Sugalski M. T., LaPrade R. F. (2006). Anatomy and biomechanics of the lateral side of the knee. *Sports Medicine and Arthroscopy Review*.

[2] Claes S., Vereecke E., Maes M., Victor J., Verdonk P., Bellemans J. (2013). Anatomy of the anterolateral ligament of the knee. *Journal of Anatomy*.

[3] Segond P. (1879). Recherches cliniques et experimentales sur les epanchements sanguine du genou par endorse. *Progrès Medical*.

[4] Hughston J. C., Andrews J. R., Cross M. J., Moschi A. (1976). Classification of knee ligament instabilities—part II: the lateral compartment. *Journal of Bone and Joint Surgery—Series A*.

[5] Johnson L. L. (1979). Lateral capsular ligament complex: anatomical and surgical considerations. *The American Journal of Sports Medicine*.

[6] Irvine G. B., Dias J. J., Finlay D. B. L. (1987). Segond fractures of the lateral tibial condyle: brief report. *Journal of Bone and Joint Surgery—Series B*.

[7] Haims A. H., Medvecky M. J., Pavlovich R., Katz L. D. (2003). MR imaging of the anatomy of and injuries to the lateral and posterolateral aspects of the knee. *American Journal of Roentgenology*.

[8] Moorman C. T., LaPrade R. F. (2005). Anatomy and biomechanics of the posterolateral corner of the knee. *The Journal of Knee Surgery*.

[9] Campos J. C., Chung C. B., Lektrakul N. (2001). Pathogenesis of the Segond fracture: anatomic and MR imaging evidence of an iliotibial tract or anterior oblique band avulsion. *Radiology*.

[10] Vieira E. L. C., Vieira E. Á., da Silva R. T., dos Santos Berlfein P. A., Abdalla R. J., Cohen M. (2007). An anatomic study of the iliotibial tract. *Arthroscopy*.

[11] Vincent J.-P., Magnussen R. A., Gezmez F. (2012). The anterolateral ligament of the human knee: an anatomic and histologic study. *Knee Surgery, Sports Traumatology, Arthroscopy*.

[12] Diamantopoulos A., Tokis A., Tzurbakis M., Patsopoulos I., Georgoulis A. (2005). The posterolateral corner of the knee: evaluation under microsurgical dissection. *Arthroscopy*.

[13] Dodds A. L., Gupte C. M., Neyret P., Williams A. M., Amis A. A. (2014). The anterolateral ligament: anatomy, length changes and association with the segond fracture. *The Bone & Joint Journal B*.

[14] El-Gharbawy R. M. (2006). Anatomy of the ligamentous and tendinous structures of the posterolateral corner of the knee. A proposal for their repair. *Bulletin of Alexandria Faculty of Medicine*.

[15] Helito C. P., Demange M. K., Bonadio M. B. (2013). Anatomy and histology of the knee anterolateral ligament. *Orthopaedic Journal of Sports Medicine*.

[16] Helito C. P., Miyahara H. S., Bonadio M. B. (2013). Anatomical study of the anterolateral knee ligament. *Revista Brasileira de Ortopedia*.

[17] Monaco E., Maestri B., Labianca L. (2010). Navigated knee kinematics after tear of the ACL and its secondary restraints: preliminary results. *Orthopedics*.

[18] http://www.abcam.com/ps/products/150/ab150686/documents/ab150686-Trichrome%20Stain%20Kit%20%28website%29.pdf

[19] Van Dyck P., Clockaerts S., Vanhoenacker F. M. (2016). Anterolateral ligament abnormalities in patients with acute anterior cruciate ligament rupture are associated with lateral meniscal and osseous injuries. *European Radiology*.

